# An investigation into the impact of family integrated care on extrauterine growth restriction at discharge in very low birth weight infants: a multi-centre study

**DOI:** 10.7189/jogh.15.04168

**Published:** 2025-08-11

**Authors:** Meng-Fan Qiu, Yi Zhang, Ying-Zi Tang, Ya-Lan Dou, Yuan Wang, Tian-Chan Lyu, Qiao-Ling Fan, Yue-Lan Ma, Fang Li, Hui Rong, Yun-Fei Tang, Wei-Wei Gu, Xiao-Chun Chen, Dan Liu, Hua Wang, Juan Xiao, Li-Li Zhang, Yan Wang, Ru-Ming Ye, Dan Li, Xiao-Xia Li, Yu Pang, Mei Lin, Mei Lin, Yan Xuan, Xiao-Jing Hu, Research Group of Developmental Care for Very Low Birth Weight Infants in China

**Affiliations:** 1 Fujian Key Laboratory of Neonatal Diseases, Xiamen Key Laboratory of Neonatal Diseases, Xiamen Children’s Hospital (Children’s Hospital of Fudan University at Xiamen), Xiamen, China; 2 Neonatal Intensive Care Unit, Children’s Hospital of Fudan University, Shanghai, China; 3 School of Nursing, Fudan University, Shanghai, China; 4 Department of Neonatology, The Affiliated Maternity and Child Health Care Hospital of Nantong University, Nantong, China; 5 Department of Neonatology, Maternal and Child Health Hospital of Guangxi Zhuang Autonomous Region, Nanning, China; 6 Department of Clinical Epidemiology and Clinical Trial Unit, Children’s Hospital of Fudan University, Shanghai, China; 7 Neonatal Intensive Care Unit, Children’s Hospital of Shanghai, Shanghai, China; 8 Neonatal Intensive Care Unit, Suzhou Municipal Hospital, Suzhou, China; 9 Pediatric Internal Medicine, Children’s Hospital of Nanjing Medical University, Nanjing, China; 10 Pediatric Department, Wuxi Children’s Hospital, Wuxi, China; 11 Neonatal Intensive Care Unit, Ningbo Women and Children’s Hospital, Ningbo, China; 12 Neonatal Intensive Care Unit, Yuying Children’s Hospital of Wenzhou Medical university, Wenzhou, China; 13 Department of Neonatology, Zhejiang Maternal Hospital, Hangzhou, China; 14 Department of Neonatology, The First Affiliated Hospital of Anhui Medical University, Hefei, China; 15 Department of Neonatology, Anhui Provincial Children’s Hospital, Hefei, China; 16 Department of Neonatology, People’s hospital of Longhua, Shenzhen, China; 17 Department of Neonatology, Yulin Maternal and Child Health Care Hospital, Yulin, China; 18 Department of Neonatology, Affiliated Hospital of Youjiang Medical College for Nationalities, Baise, China; 19 Department of Neonatology, Qinzhou Maternal and Child Health Hospital, Qinzhou, Guangxi, China; 20 Department of Nursing, Hainan Women and Children’s Medical Centre, Haikou, China; 21 Department of Nursing, Children’s Hospital of Fudan University, Shanghai, China; 22 Research Unit of Early Intervention of Genetically Related Childhood Cardiovascular Diseases (2018RU002), Chinese Academy of Medical Sciences, Shanghai, China

## Abstract

**Background:**

Family integrated care (FIC) encourages parental involvement in neonatal intensive care units (NICU) and has been found to promote weight gain in preterm infants. Extrauterine growth restriction (EUGR) results from inadequate growth among very low birth weight infants (VLBWI), which has been found to contribute to parental anxiety. To address an existing gap in research, we aimed to examine the impact of parental involvement on EUGR at discharge in VLBWI.

**Methods:**

We conducted a retrospective, multi-centre case-control study involving VLBWIs admitted to 17 NICUs across eight southeastern Chinese provinces and cities from February 2021 to November 2023. We categorised cases and control groups based on the presence of EUGR at discharge and compared their perinatal and hospitalisation characteristics, as well as FIC duration, using a generalised linear mixed model.

**Results:**

EUGR in VLBWI at discharge was associated with birth weight (odds ratio (OR) = 0.547; 95% confidence interval (CI) = 0.490, 0.610), gestational week (<28 weeks) (OR = 3.101; 95% CI = 1.909, 5.038), Apgar score at 1 minute ≤7 (OR = 1.525; 95% CI = 1.119, 2.079), being small for gestational age (OR = 3.269; 95% CI = 1.547, 6.908), maternal gestational hypertension (OR = 1.868; 95% CI = 1.270, 2.748), necrotising enterocolitis (OR = 2.254; 95% CI = 1.386, 3.667), and total FIC duration. Based on literature and clinical practice, we divided the total FIC duration into three groups. We found that the lowest OR was associated with >18 hours of care, followed by ≤18 hours, while the highest was associated with 0 hours of care.

**Conclusions:**

We identified higher birth weight and FIC as protective factors against EUGR at discharge in VLBWI. In contrast, we recognised gestational age <28 weeks, an Apgar score ≤7 at 1 minute, small for gestational age, maternal gestational hypertension, and necrotising enterocolitis as risk factors. Nevertheless, further research is required to analyse the relationship between FIC and EUGR at discharge.

**Registration:**

ClinicalTrials.gov (NCT06550440).

Very low-birth-weight infants (VLBWI), defined as live newborns weighing less than 1500 g, represent a significant proportion of patients in neonatal intensive care units (NICU). According to the 2022 annual report from the Chinese Neonatal Network, the survival rate for VLBWI in China is approximately 87.9%. However, due to their low birth weight and immature organ development, weight gain has become a critical concern regarding their survival. A recent multi-centre study in China reported an extrauterine growth restriction (EUGR) incidence of 60.7% at discharge among VLBWI [1], while a European cohort study found rates ranging from 24% to 60% [2]. This high incidence in China could be related to delays in achieving full enteral feeding that exceed international guideline recommendations [1,3]. Notably, EUGR adversely affects the long-term growth and development of VLBWI after discharge. Takayanagi and colleagues reported associations of EUGR with short stature, and lower body weight in preschool-aged children born as VLBWI [4], while other studies also linked it to impaired neurodevelopment with reduced mental development indices at two years [5,6], and lower intelligence quotient scores at five years [7]. Additionally, EUGR may contribute to adverse metabolic outcomes, including elevated blood glucose in prepubertal children [8]. Therefore, it is vital to identify and prevent the influencing factors of EUGR in VLBWI.

Previous research has focussed primarily on nutritional status [9] and clinical complications [10]. Zhang and colleagues found that higher levels of maternal posttraumatic stress, often accompanied by anxiety and depression, were associated with growth retardation in preterm infants [11], highlighting the importance of parental factors. Family Integrated Care (FIC), introduced by Shoo Lee in Canada, encourages parental involvement in non-medical care in the NICU [10], and has been shown to reduce maternal anxiety and caregiving stress [10,12]. Since its effect on EUGR at discharge in VLBWI remains unclear, we explored the impact of FIC on EUGR to support the early identification of EUGR and inform interventions targeted at improving weight gain among affected populations. We hypothesised that FIC may reduce EUGR incidence and enhance growth outcomes in VLBWI.

## METHODS

### Study design

We conducted a multi-centre retrospective case-control study, categorising case and control groups based on the occurrence of EUGR at discharge in order to explore whether FIC was associated with EUGR at discharge in VLBWI. We followed the STROBE guidelines in reporting our findings (Checklist S1 in the **Online Supplementary Document**).

### Study setting and samples

We carried out the study at 17 units within tertiary hospitals across eight provinces (cities) in southeastern China: Jiangsu (three hospitals), Shanghai (two hospitals), Zhejiang (three hospitals), Anhui (two hospitals), Fujian (one hospital), Guangdong (one hospital), Guangxi (four hospitals), and Hainan (one hospital). All involved hospitals serve as treatment centres for critically ill newborns.

Based on the inclusion of approximately 26 variables and a requirement to have at least 10 cases per variable in regression analysis [13,14], as well as an estimated EUGR incidence of 60.7% [1], we calculated that we would require a sample size of 428 participants. We included neonates who met the following conditions: birth weight <1500 g and born <32 weeks of gestation; admitted to the NICU within 24 hours after birth; on the day of admission there were no complications related to preterm birth, involving bronchopulmonary dysplasia (BPD), retinopathy of prematurity (ROP), necrotising enterocolitis (NEC), and intraventricular haemorrhage (IVH). Conversely, we excluded neonates who died in the NICU or had incomplete outcome data, regardless of whether they were discharged as VLBWI with or without EUGR.

### Data collection

We retrieved clinical data from the unified database of the Research Group of Developmental Care for Very Low Birth Weight Infants in China, which included: perinatal characteristics of newborns (sex, gestational weeks at birth, birth weight, mode of delivery, multiparity, in vitro fertilisation, Apgar score at 1 minute ≤7, and whether they wer esmall for gestational age (SGA)); length of stay in NICU; major complications encountered in NICU (excluding the day of admission): incidence of BPD, ROP, NEC, IVH and late-onset sepsis (LOS); nutritional support provided to VLBWI – duration of parenteral nutrition, breastfeeding days, rate of feeding on their own mothers’ breasts, oral immunotherapy and oral motor intervention (OMI); the total duration of FIC; maternal perinatal characteristics (advanced maternal age (>35 years), gestational diabetes mellitus, gestational hypertension, antenatal corticosteroids use, antenatal magnesium sulphate use, and antenatal antibiotic use).

We categorised the total duration of FIC for VLBWI during hospitalisation as a multi-categorical variable based on hours. Literature suggests that parents spend at least six hours caring for infants in NICUs [10,12]. Moreover, many real-world NICUs invite parents to participate in three-day, six-hour caregiving training sessions, which is also the case with NICUs in China the initial stage of FIC implementation. Therefore, we set 18 hours (six hours per day over three days) as a threshold to classify FIC duration into three categories: 0 hours, ≤18 hours, and >18 hours.

Each hospital appointed data quality control specialists to organise and input data, with every entry double-checked by two individuals. The group leader trained all specialists and conducted quarterly data reviews to ensure accuracy and integrity.

### Study definitions and diagnostic criteria

We defined key clinical terms and criteria used in the study as follows:

− EUGR: weight at discharge ≤10th percentile for sex and gestational age, based on infants born during the same period [15,16], using the Fenton growth curve from 2013 [17].
− SGA: birth weight <10th percentile for sex and gestational age [18,19].
− BPD: oxygen required at 36 weeks corrected gestational age or discharge [20].
− ROP: diagnosed per the International Classification of Retinopathy of Prematurity [21].
− NEC: diagnosed according to the Bella classification [22].
− IVH: diagnosed based on Paplie’s classification [23].
− LOS: sepsis occurring >72 hours after birth [24].
− OMI: stimulating the perioral and intraoral regions, followed by pacifier or finger use for non-nutritive sucking before feeding [25].
− FIC: trained NICU staff educate parents to actively care for their infants, providing non-medical care like feeding, diaper changes, and skin-to-skin contact [10]. This approach allows flexible parental involvement once the infant is stable, with varying daily participation time. The total duration of FIC is defined as the cumulative hours of parental involvement from entry to exit in the NICU.

### Statistical analyses

We expressed skewed continuous variables as medians with interquartile ranges (IQR) and compared them using non-parametric tests. We presented categorical variables as frequencies and proportions, utilising χ^2^ tests for group comparisons. Additionally, we used PASS, version 15.0 (NCSS, LLC, Kaysville, UT, USA) to calculate the statistical power of this study. We imputed missing variables using the multiple imputation method.

We used a generalised linear mixed model (GLMM) to estimate odds ratios (OR) and 95% confidence intervals (CI) for associations between perinatal variables and EUGR at discharge, accounting for hospital-level effects. The presence or absence of EUGR at discharge was used as the dependent variable to construct the model. We included variables with significant differences at baseline (*P* < 0.1) or with clinical significance into the model. Using *P* < 0.1 as a univariate screening criterion can improve the robustness of the multivariable model and minimise the risk of excluding potential predictive variables [26]. We determined the covariance among the included variables using the variance inflation factor, including variables without covariance in fixed effects, hospitals in random effects, and conducting pairwise contrasts for categorical variables within the variables. A *P*-value of <0.05 was considered a statistically significant difference.

We conducted statistical analyses using SPSS, version 27.0 (IBM Corp, Armonk, NY, USA) all generated all visualisations using *R*, version 4.3.2 (R Core Team, Vienna, Austria). We also used *R* and its ‘mice’ package for the multiple imputation.

## RESULTS

**Figure 1:**
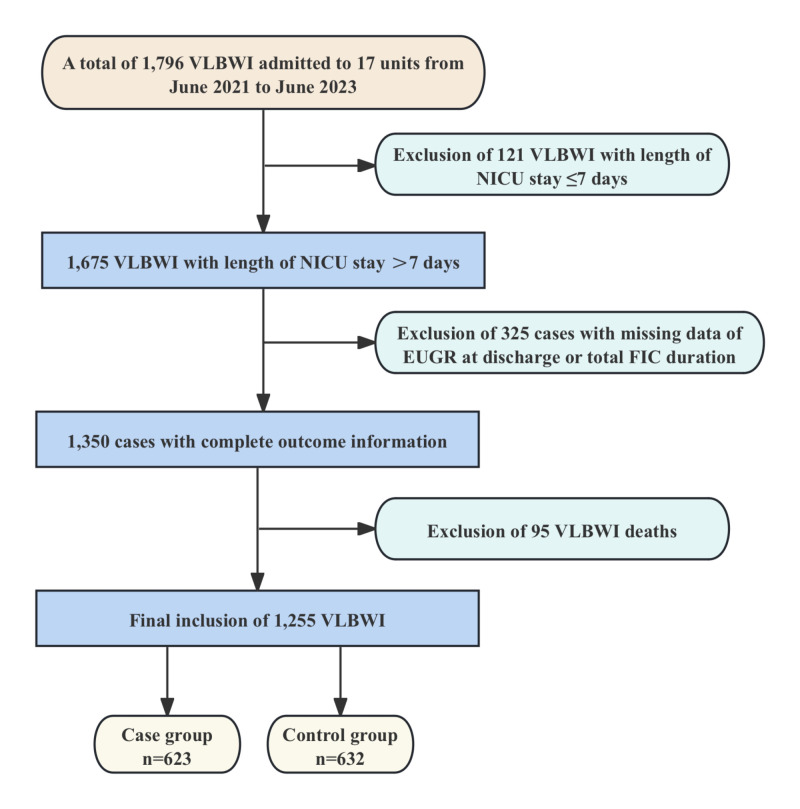
Flowchart for study population selection. EUGR – extrauterine growth restriction, FIC – family integrated care, NICU – neonatal intensive care unit, VLBWI – very low birth weight infants.

**Figure 2:**
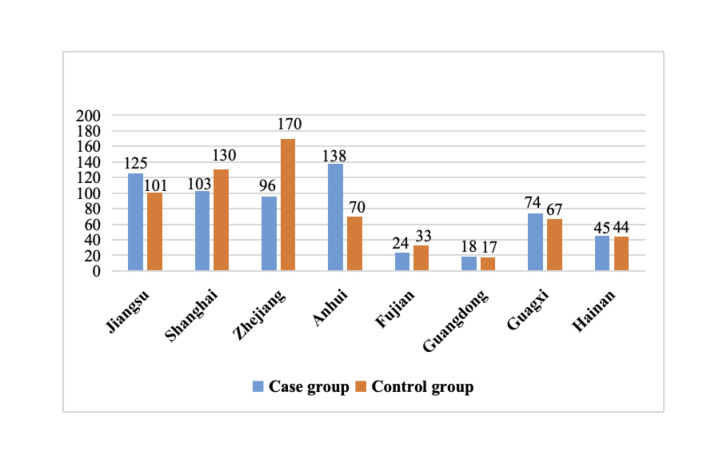
Number of study population in case and control groups by province (city).

We analysed 1255 VLBWI cases VLBWI from 17 hospitals (excluding 121 cases with length of NICU stay ≤7 days, 325 cases with missing outcome variables of EUGR at discharge or total FIC duration, and 95 cases of death before grouping), with 623 (49.6%) in the case group and 632 (50.4%) in the control group (**Figure 1**). We calculated the study power to be >0.995, indicating that we had a sufficient sample size (**Figure 2**).

The following variables had missing data and were addressed through multiple imputation: Apgar score at 1 minute ≤7 (2.3%), maternal age >35 years (3.9%), BPD, ROP, and IVH (each 0.2%), NEC (0.4%), LOS (0.6%), gestational diabetes and hypertension (each 1.5%), antenatal corticosteroid, magnesium sulphate, and antibiotic use (each 3.9%), parenteral nutrition days (5.3%), breastfeeding days (7.9%), and OMI (1.0%).

We observed differences between the case and control groups in several variables, with all having *P*-values <0.1: male sex, birth weight, mode of delivery (caesarean section), Apgar score at 1 minute ≤7, SGA, maternal gestational hypertension, maternal use of antenatal antibiotics, length of stay in the NICU, NEC, LOS, IVH, breastfeeding duration in days, OMI, and total duration of FIC (**Table 1**).

**Table 1:** Baseline characteristics of the study population*

	Case group (n = 623)	Control group (n = 632)	χ^2^/z	*P*-value
**Newborn characteristics**				
Male sex	309 (49.6)	356 (56.3)	5.705†	0.017
**Gestational weeks**				
*<28*	78 (12.5)	96 (15.2)	1.873†	0.171
*28 ~ 31^+6^*	545 (87.5)	536 (84.8)		
**Birth weight, per 100 g, MD (IQR)**	11.3 (9.9, 12.8)	12.7 (11.4, 13.9)	−11.620‡	<0.001
**Caesarean delivery**	434 (69.7)	396 (62.7)	6.873†	0.009
**Multiple births**	204 (32.7)	188 (29.7)	1.313†	0.252
**In vitro fertilisation**	129 (20.7)	145 (22.9)	0.920†	0.338
**Apgar score at 1minute ≤7**	285 (45.7)	192 (30.4)	32.442†	<0.001
**SGA**	77 (12.4)	10 (1.6)	56.481†	<0.001
**Maternal characteristics**				
Maternal age >35 y	122 (19.6)	129 (20.4)	0.135†	0.714
Gestational diabetes mellitus	145 (23.3)	151 (23.9)	0.066†	0.797
Gestational hypertension	174 (27.9)	83 (13.1)	42.179†	<0.001
Antenatal corticosteroids	457 (73.4)	459 (72.6)	0.084†	0.771
Antenatal magnesium sulphate	264 (42.4)	276 (43.7)	0.215†	0.643
Antenatal antibiotics	186 (29.9)	234 (37.0)	7.243†	0.007
**NICU stay period**				
Length of stay in days, MD (IQR)	59.0 (47.0, 72.0)	55.5 (46.0, 68.0)	−2.774‡	0.006
**Major complications**				
BPD	278 (44.6)	307 (48.6)	1.970†	0.160
ROP	181 (29.1)	159 (25.2)	2.409†	0.121
NEC	89 (14.3)	48 (7.6)	14.442†	<0.001
IVH	191 (30.7)	165 (26.1)	3.197†	0.074
LOS	165 (26.5)	101 (16.0)	20.723†	<0.001
**Nutrition support**				
Parenteral nutrition in days, MD (IQR)	13.0 (0.0, 25.0)	14.0 (0.0, 25.0)	−0.576‡	0.564
Breastfeeding days in days, MD (IQR)	9.0 (0.0, 37.0)	25.5 (0.0, 46.0)	−4.992‡	<0.001
Feeding on their own mothers’ breasts	72 (11.6)	65 (10.3)	0.522†	0.470
Oral immunotherapy	202 (32.4)	225 (35.6)	1.411†	0.235
OMI	273 (43.8)	344 (54.4)	14.131†	<0.001
**Duration of FIC in hours**				
*>18*	97 (15.6)	142 (22.5)	−5.166‡	<0.001
*~ 18*	66 (10.6)	115 (18.2)		
*0*	460 (73.8)	375 (59.3)		

BPD – bronchopulmonary dysplasia, CI – confidence interval, FIC – family integrated care, IVH – intraventricular haemorrhage, LOS – late-onset sepsis, NEC – necrotising enterocolitis, OMI – oral motor intervention, OR – odds ratio, ROP – retinopathy of prematurity, SGA – small for gestational age

*Presented as n (%) unless specified otherwise.

†χ^2^ test.

‡Non-parametric test.

We analysed all 14 variables for covariance, with variance inflation factor of <10 and no covariance between variables. We took into account the clinical significance of gestational age and included it alongside the 14 variables in our analysis (**Table 2**). The results indicated that protective factors for the occurrence of EUGR at discharge in VLBWI included larger birth weight and FIC (*P* < 0.001). Conversely, risk factors comprised gestational age less than 28 weeks (*P* < 0.001), an Apgar score at 1 minute of ≤7 (*P* < 0.05), SGA(*P* < 0.05), maternal gestational hypertension (*P* = 0.002), and NEC(*P* < 0.001).

**Table 2:** Analysis of factors influencing the occurrence of extrauterine growth restriction in very low birth weight infants at discharge

	Before multiple imputation		After multiple imputation	
**Variable**	**OR (95% CI)**	***P*-value**	**OR (95% CI)**	***P*-value**
Male sex	1.157 (0.858, 1.562)	0.339	1.129 (0.845, 1.507)	0.412
Birth weight, per 100 g	0.553 (0.494, 0.620)	<0.001	0.547 (0.490, 0.610)	<0.001
Gestational week				
*<28*	2.975 (1.798, 4.922)	<0.001	3.101 (1.909, 5.038)	<0.001
*28 ~ 31^+6^*				
Caesarean delivery	1.231 (0.888, 1.707)	0.211	1.296 (0.946, 1.775)	0.106
Apgar score at 1 min ≤7	1.504 (1.092, 2.072)	0.012	1.525 (1.119, 2.079)	0.008
SGA	2.763 (1.290, 5.918)	0.009	3.269 (1.547, 6.908)	0.002
Gestational hypertension	1.887 (1.267, 2.812)	0.002	1.868 (1.270, 2.748)	0.002
Antenatal antibiotics	0.854 (0.603, 1.210)	0.376	0.864 (0.618, 1.209)	0.394
Length of stay in days	0.930 (0.853, 1.013)	0.098	0.992 (0.984, 1.000)	0.064
NEC	2.580 (1.545, 4.308)	<0.001	2.254 (1.386, 3.667)	0.001
LOS	1.190 (0.810, 1.749)	0.375	1.155 (0.795, 1.679)	0.450
IVH	1.217 (0.833, 1.778)	0.310	1.231 (0.851,1.780)	0.270
Breastfeeding days	1.002 (0.994, 1.010)	0.612	1.001 (0.993, 1.008)	0.870
OMI	0.973 (0.660, 1.434)	0.891	0.918 (0.634, 1.329)	0.650
Total Duration of FIC in hours				
*>18*	0.180 (0.106, 0.307)	<0.001	0.166 (0.100, 0.275)	<0.001
*~ 18*	0.395 (0.232, 0.673)	0.001	0.360 (0.216, 0.602)	<0.001
*0 (reference)*				

CI – confidence interval, FIC – family integrated care, LOS – late-onset sepsis, NEC – necrotising enterocolitis, OMI – oral motor intervention, OR – odds ratio, SGA – small for gestational age

**Figure 3:**
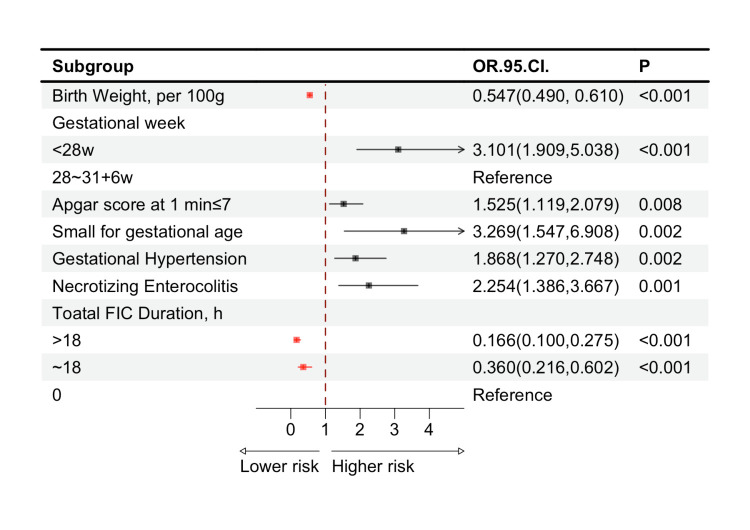
Factors influencing extrauterine growth restriction in very low birth weight infants at discharge. CI – confidence interval, FIC – family integrated care, OR – odds ratio.

We found that, compared to VLBWI without FIC (0 hours), those receiving FIC for ≤18 hours and >18 hours had significantly reduced risks of EUGR at discharge, indicating a 64.0% (OR = 0.360; 95% CI = 0.216, 0.602; *P* < 0.001) and 83.4% (OR = 0.166; 95% CI = 0.100, 0.275; *P* < 0.001) risk reduction, respectively. In pairwise analyses, a total FIC duration of ≤18 hours exhibited a smaller OR for EUGR at discharge compared to a total FIC duration of 0 hours (*P* = 0.001). In contrast, a total FIC duration exceeding 18 hours demonstrated a reduced OR for EUGR at discharge when compared to a total FIC duration of ≤18 hours (*P* = 0.005). Furthermore, the OR for EUGR at discharge was significantly lower when comparing FIC duration greater than 18 hours and an FIC of 0 hours (*P* < 0.001) (**Figure 3****,** **Table 3**).

**Table 3:** Pairwise contrasts of FIC hours

	Before multiple imputation		After multiple imputation	
**Total duration of FIC in hours**	**OR (95% CI)**	***P*-value**	**OR (95% CI)**	***P-*value**
≤18 *vs. *0	−0.203 (−0.335, −0.070)	0.003	−0.129 (−0.351, −0.087)	0.001
>18 *vs.* ≤18	−0.193 (−0.329, −0.057)	0.005	−0.191 (−0.322, −0.059)	0.005
>18 *vs.* 0	−0.396 (−0.520, −0.271)	<0.001	−0.410 (−0.531, −0.288)	<0.001

CI – confidence interval, FIC – family integrated care, OR – odds ratio

## DISCUSSION

Definitions of EUGR vary across studies and are generally categorised as cross-sectional or longitudinal [27]. The former relies on a z-score<−2 or an anthropometric indicator ≤10th percentile at 36–40 weeks corrected gestational age or at discharge [15,28], while the latter refers to a weight loss ≥1 or 2 standard deviations from the mean, measured from birth to discharge or 36 weeks corrected age [28,29]. However, percentiles may be less precise in quantifying deviation from the mean compared to z-scores. Given the multi-centre nature of this study and standardised training across sites, we adopted the weight-based cross-sectional definition for consistency. The GLMM results remained stable before and after multiple imputation, supporting the robustness of our findings.

We determined gestational age based on the mother’s self-reported last menstrual period, which may have introduced subjectivity. Therefore, we grouped gestational age into two categories: <28 weeks (extremely preterm) and 28–31^+6^ weeks (very preterm). We found no significant difference in gestational age between the case and control groups. However, given its clinical relevance, we still included it in the analysis. The results indicated that gestational age <28 weeks increases the risk of EUGR at discharge (OR = 3.101; 95% CI = 1.909, 5.038), likely due to the immaturity of organs and underdeveloped gastrointestinal function in extremely preterm VLBWI.

Research have shown that SGA in preterm infants often results from a combination of maternal, placental, and foetal effects [30]. For example, mothers with gestational hypertension are more likely to have SGA [31]. In this study, the incidence of SGA and gestational hypertension was significantly higher in the case group (*P* < 0.001) (**Table 1**), and both emerged as risk factors for EUGR (*P* = 0.002) (**Table 2**). Gutbrod and colleagues [32] reported that VLBWI with SGA face increased EUGR risk due to shorter gestation and intrauterine growth restriction. Khasawneh and colleagues’ retrospective study of 247 VLBWI found SGA to be significantly associated with EUGR at discharge (adjusted OR = 9.00; 95% CI = 2.00, 50.00) [33], which aligns with findings from a single-centre study in China [34]. Yazici and colleagues also identified SGA as a major risk factor (OR = 19.15; 95% CI = 4.40, 82.59), though they defined EUGR by a discharge weight z-score<−2 [35]. These findings suggest that SGA is a reliable predictor of EUGR under the cross-sectional definition. In our analysis, SGA had the highest OR among all risk factors (**Figure 3**). This points to a need to improve nutritional management during hospitalisation for SGA in VLBWI to promote catch-up growth. Demg and colleagues [36] identified both VLBWI (OR = 5.5; 95% CI = 2.1, 14.8) and SGA (OR = 4.8; 95% CI = 1.8, 12.8) as risk factors for poor growth in the first year of life. Consequently, SGA in VLBWI requires continuous and intensive nutritional support during hospitalisation and after discharge.

Preterm infants with an Apgar score of ≤7 at 1 minute are considered to be at risk of having experienced birth asphyxia. Dolgun and colleagues [37] indicated that the Apgar score at 1 minute after birth correlates positively with birth weight. Xiang and colleagues found that the case group had a higher percentage of Apgar score ≤7 at 1 minute and lower birth weight compared to the control group (*P* < 0.05). Since birth weight is the basis for weight gain in VLBWI, lower birth weight is likely to be a risk factor for EUGR at discharge [38].

In addition to perinatal factors, complications during hospitalisation also impact weight gain in VLBWI. Conditions such as impaired intestinal barrier function, gut flora imbalance, and improper enteral feeding can lead to NEC. Here we identified NEC to be a risk factor for EUGR at discharge, which aligns with the multi-centre study by Yu and colleagues [1]. We note that NEC may require interventions like fasting or surgery [22]. One study [39] suggests that breast milk and probiotics have a synergistic effect in reducing NEC incidence, with meta-analyses showing breast milk protects against NEC [40], while the latter has also been found to significantly reduce NEC morbidity and mortality in VLBWI [41,42].

Our findings suggest that greater birth weight was a protective factor for EUGR at discharge in VLBWI. This is consistent with Makker and colleagues’ prospective cohort study [43], which included 1063 preterm infants and found an association between higher birth weight and lower risk of EUGR at discharge (OR = 0.99; 95% CI = 0.99, 0.99). We also included the birth weight per 100 g in the model, which resulted in a lower OR value.

Most studies examining factors that influence EUGR at discharge have primarily focussed on neonatal characteristics. Recently, changing attitudes have led to increased parental involvement in the care of preterm infants in the NICUs. Several studies have shown that FIC, which includes practices like kangaroo care and breastfeeding, can improve daily weight gain during hospitalisation [10,12]. However, the impact of FIC on EUGR at discharge in VLBWI has not been well studied.

One pilot study [10] and a multi-centre cluster randomised controlled trial study [12] showed that preterm infants in the FIC group had a faster average daily weight gain during hospitalisation than the control group (*P* < 0.01). A Chinese cluster randomised controlled trial reported similar findings [44], while a multi-centre study found that a faster average daily rate of weight gain was a protective factor for EUGR [45]. We therefore hypothesised that FIC might be a protective factor for EUGR, which was confirmed by our GLMM analysis.

In general, FIC is a flexible care model that has varying levels and durations of parental involvement, which is likely to affect its outcomes. Some high-quality cluster randomised controlled trials [12,44] reported FIC duration as ≥6 hours/d, but the total hours of parental care in the NICU remain unstandardised. Most hospitals in our group employ a three-day FIC model alongside clinical practice. Therefore, we categorised FIC duration into three groups using 18 hours (3 × 6 hours) as the cutoff to assess its impact on EUGR at discharge in VLBWI. We observed the lowest OR for EUGR in infants receiving >18 hours of FIC, followed by those receiving ≤18 hours, and the highest in those without FIC (**Table 3**). However, more studies are needed on how the effect of FIC differs depending on its length. FIC may promote parental participation in practices such as kangaroo care and feeding, both of which have been shown to enhance weight gain and reduce feeding intolerance in premature infants. This approach may further contribute to a lower risk of EUGR by fostering stronger emotional bonds between parents and infants [46,47]. Furthermore, under the FIC model, parents are more likely to prefer single-family rooms (one room with one bed) or double-occupancy rooms (one room with two beds), which help minimise environmental noise and create a more conducive setting for infant sleep and development [48].

Breastfeeding is an important component of FIC. Increasing the amount of breastfeeding has been reported to have a positive effect on VLBWI weight gain [49]. Interestingly, we found that the duration of breastfeeding did not affect EUGR at discharge (*P* = 0.870), possibly due to variations in daily intake volume and use of human milk fortifier. Previous research suggests that a daily intake of ≥50 ml/kg of breast milk in the first four weeks is linked to lower EUGR incidence [50]. Furthermore, since the protein and energy requirements of VLBWI exceed those of healthy full-term newborns, breast milk alone cannot fulfil the nutritional needs that VLBWI typically require. Human milk fortifiers are typically introduced when daily feedings reach 80 ~ 100 ml/kg and have been shown to enhance weight gain during hospitalisation [51]. Guidelines also recommend multicomponent fortification to support preterm growth [52]. In addition, Wang and collagues [45] found that later addition of human milk fortifier was a risk factor for the development of EUGR in preterm infants (OR = 1.022; 95% CI = 1.012, 1.032). As a retrospective study, we did not collect data on daily breast milk volume or fortifier use. Further prospective research is necessary to clarify these associations.

Targeted nutritional management is essential for SGA infants, those with lower birth weight and gestational age, as well as infants with birth asphyxia or maternal gestational hypertension. Preventing NEC is also critical in reducing EUGR risk. While considering the available human resources, educating parents to increase their involvement in care could positively impact the reduction of EUGR incidence.

### Study limitations

Some of the limitations of this study are our exclusive enrollment of VLBWI from southeastern China, which may have introduced selection bias. We used the cross-sectional definition of EUGR, with body weight as the sole diagnostic indicator, which limits the generalisability of our findings.

## CONCLUSIONS

In this retrospective study, we examined factors associated with EUGR at discharge in VLBWI using a cross-sectional definition and found that higher birth weight and FIC were protective factors. We categorised the duration of FIC into three groups and observed that the smallest OR for EUGR was associated with a care duration exceeding 18 hours, followed by duration less than 18 hours, and finally, 0 hours of FIC provided. This study is novel in exploring the relationship between FIC duration and EUGR, offering a parental care perspective. Although existing evidence has highlighted the benefits of FIC, many countries or regions have yet to implement it. Our findings may encourage incremental progress in neonatal care practices. We concluded that even if full implementation of FIC is not feasible, achieving at least 18 hours of FIC still benefits the growth of VLBWI. However, our findings might be limited by selection bias, and future studies with wider regional coverage and high-quality designs are necessary to clarify the dose-response effect between FIC duration and EUGR.

## Additional material


Online Supplementary Document
